# Association between medicated obstructive pulmonary disease, depression and subjective health: results from the population-based Gutenberg Health Study

**DOI:** 10.1038/s41598-019-56440-9

**Published:** 2019-12-27

**Authors:** Jasmin Ghaemi Kerahrodi, Elmar Brähler, Jörg Wiltink, Matthias Michal, Andreas Schulz, Phillip S. Wild, Thomas Münzel, Gerrit Toenges, Karl J. Lackner, Norbert Pfeiffer, Manfred E. Beutel

**Affiliations:** 1 Department of Psychosomatic Medicine and Psychotherapy, University Medical Center of the Johannes Gutenberg University Mainz, Mainz, Germany; 2 Center for Cardiology, Cardiology I, University Medical Center of the Johannes Gutenberg University Mainz, Mainz, Germany; 3 Preventive Cardiology and Preventive Medicine, Center for Cardiology, University Medical Center of the Johannes Gutenberg-University Mainz, Mainz, Germany; 4 Center for Thrombosis and Hemostasis, University Medical Center of the Johannes Gutenberg-University Mainz, Mainz, Germany; 5 DZHK (German Center for Cardiovascular Research), partner site RhineMain, Mainz, Germany; 6 Institute for Clinical Chemistry and Laboratory Medicine, University Medical Center of the Johannes Gutenberg University Mainz, Mainz, Germany; 7 Institute for Medical Biostatistics, Epidemiology and Informatics, University Medical Center of the Johannes Gutenberg-University Mainz, Mainz, Germany; 8 Department of Ophthalmology, University Medical Center of the Johannes Gutenberg-University Mainz, Mainz, Germany

**Keywords:** Diseases, Risk factors

## Abstract

Medicated obstructive pulmonary disease (asthma or COPD) has been associated with depression. Yet, there is little knowledge of the interplay of contributing social, biological, behavioral and psychological factors in the community. The study was conducted: (1) To determine the prevalence of depression in participants with medicated COPD or asthma from the general population, (2) to identify underlying social, biological, behavioral and psychological factors and (3) to determine the contribution of obstructive pulmonary disease and depression to subjective health. The population-based sample of 15.010 study participants (35–74 years) from the Gutenberg Health Study (GHS) was queried according to a medical diagnosis of obstructive pulmonary disease, defined as medicated COPD or asthma, and comorbid disorders. Demographic, behavioral and psychological factors were assessed by self-report; lung function (FEV1; FCV) was measured by spirometry. 307 men (4.3%) and 396 women (5.6%) reported a medical diagnosis of COPD or asthma. The prevalence of depression (PHQ-9 > = 10) was twice as high (16.2% vs. 7.5%) compared to participants without obstructive pulmonary disease. Participants with obstructive pulmonary disease were older, had a lower SES, more comorbid diseases and cardiovascular risk factors, higher distress and took more psychotropic medication. In multivariable logistic regression analyses, obstructive pulmonary disease was associated with a 71% increase of depression (OR = 1.71; 95% CI = 1.30 to 2.24). Additional contributors were FEV1 (1.18; 95% CI = 1.05 to 1.32) and dyspnea (NYHA > = 1) (2.19; 1.82 to 2.64), sex (women) (OR = 1.73; 95% CI 1.41 to 2.12), lower SES (OR = 0.98; 95%CI = 0.96 to 0.99). Lack of active sports OR = 0.79; 95% CI 0.68 to 0.92), obesity (OR 1.27; 95% CI 1.07 to 1.50), smoking (OR = 1.26; 95% CI 1.06 to 1.49) and dyslipidemia (OR = 1.35; 95% CI 1.15 to 1.57) also increased the risk of depression. Additional psychological risks were social phobia, type D, low social support, loneliness and life events in the past 12 months. In multivariable linear regression analyses, obstructive pulmonary disease and depression independently contributed to reduced subjective health in addition to sedentary behavior, smoking and comorbid somatic and mental disorders. These findings provide evidence that COPD and asthma are associated with depression in the community. Complex underlying demographic, medical and psychosocial variables have been identified which may justify an integrative treatment approach. Promoting health behavior (smoking cessation, exercising, weight reduction) and social integration may not only improve the somatic course of the disease, but also mental health. Mental health treatment may also improve health behavior and subjective health.

## Introduction

Chronic obstructive pulmonary disease (COPD) is a major cause of worldwide morbidity and mortality. It is a complex, heterogeneous and multicomponent disease, characterized by airflow limitation, chronic systemic inflammation and progressing fibrosis with remodeling and narrowing of the small airways and destruction of the lung parenchyma^1^. Multiple somatic, esp. cardiovascular and mental comorbidities have been described^2,3^ affecting clinical severity and prognosis^4,5^. Risk factors overlap between COPD and other diseases, especially cardiovascular risk factors CVRFs^5^, most prominent is smoking^6^.

While depression has been considered one of the most frequent comorbidities in COPD, prevalence estimates have ranged from 18.8%^7^ to 51.5%^8^. Somatic (cardiovascular, diabetes) and mental comorbidities (depression, anxiety) were negatively associated with health-related quality of life (HRQOL) and health outcomes in patients with COPD^9–11^, increased health resource use^12^ and mortality^10,13,14^.

Associations of asthma with psychological factors have been investigated. Individuals with asthma have reported reduced health-related quality of life^15^. There is a wide variation of the prevalence of depressive symptoms in individuals with asthma ranging from 1% to 45%^16^.

Dyspnea is one of the most common symptoms in pulmonary and cardiovascular medicine. Patients with chronic diseases that lead to impaired lung function and dyspnea, are likely to have more depressive symptoms compared with healthy individuals^17^. Most likely, mechanisms work both ways^13^: obstructive pulmonary diseases such as COPD and asthma increase the risk for mental disorders^18^ for biological (dyspnea, chronic inflammation) and psychosocial reasons. On the other hand, mental distress increases the risk of smoking, the primary risk factor for COPD^6,19^. Conversely, smoking has been shown a risk factor for new onset of depression in middle-aged adults^20^. Also, there may be an influence of emotional states on lung function. Negative mood states were associated with a reduction in FEV1 compared with neutral states of emotion^21^. Ritz *et al*.^22^ found an increased total respiratory resistance in subjects with asthma following exposure to depressing stimuli.

In a comprehensive approach we analyzed the relationships^3^ of social, biological, and psychological variables with COPD or asthma and depression in a large community sample. Thus, analyses covered participants who reported that they were diagnosed and treated with COPD or asthma by a physician (and not limited to those who seek health care as in most previous studies). Yet, we were able to include a comprehensive range of biological variables including pulmonary function, symptoms (dyspnea) and comorbid disorders, social and psychological variables. Aims of the study were 1): To determine the prevalence of depression in participants with medicated COPD or asthma in the general population, and 2) to identify underlying associations of social, biological, behavioral and psychological factors and 3) to determine the contribution of obstructive pulmonary disease and depression to subjective health.

We expected an increased prevalence of depression among participants with obstructive pulmonary disease (COPD or Asthma) and a higher proportion of depression in participants with a) worse lung function as evidenced by FEV1 and dyspnea, b) additional somatic disease (CVD, cancer, etc.), c) stress (life events, loneliness) and Type D behavior. We further expected lower subjective health in participants suffering from both COPD or asthma and depressive symptoms.

## Material and Methods

### Participants and recruitment

The analysis presents data of a large-scale population-based sample. 15.010 study participants were enrolled in the Gutenberg Health Study (GHS) between the years of 2007 and 2012. The prospective, population-based, observational single-center cohort study of the University Medical Center Mainz is conducted in the Rhine-Main-Region of Western Germany. The design and the rationale of the Gutenberg Health Study (GHS) have been described in detail elsewhere^23^. This report is based on cross-sectional analyses^24^. Participants were randomly drawn from the local registry of the city of Mainz and the county of Mainz-Bingen. The study protocol and documents were approved by the ethics committee of the Medical Chamber of Rhineland-Palatinate and the local data safety commissioner. All study investigations have been conducted in line with the Declaration of Helsinki and principles outlined in recommendations for Good Clinical Practice and Good Epidemiological Practice. Accordingly, written informed consent was obtained from all participants prior to entering the study. Inclusion criterion was age between 35 and 75 years. Exclusion criteria were insufficient knowledge of German language and inability to participate due to physical or mental impairment. The sample was stratified 1:1 for gender and residence (rural and urban) and in equal strata for decades of age. Out of a sample of 28,533, 52.61% participated (N = 15,010; 53.3% of the men and 51.9% of the women). Five point two percent fulfilling exclusion criteria were excluded. Of the non-responders, 22.6% refused participation, and another 20% could not be contacted.

### Computer-assisted personal interview

Prevalent cardiovascular risk factors and other clinical variables were collected by a computer-assisted personal interview during the 5-hour baseline-examination in the study center. Blood pressure, anthropometric measurements and laboratory examinations from a venous blood sample were performed by certified medical technical assistants according to standard operating procedures.

The widely used Patient Health Questionnaire (PHQ-9) was used for screening the presence and severity of depressive disorder. It has been proven a reliable measure of depression^25^. The PHQ-9 scores each of the 9 DSM-IV criteria as “0” (not at all), “1” (several days), “2” (more than half of the days) and “3” (nearly every day). A PHQ-9 sum score ≥10 was defined as probable caseness. Lowe *et al*. found a sensitivity of 81% and a specificity of 82% for depressive disorder determined by this cut-off^25^.

Suicidal thoughts were measured by the single item (PHQ-9) “in the last two weeks, have you had thoughts that you would be better off dead or hurting yourself in some way?”.

We assessed generalized anxiety with the two item short form of the GAD-7 (Generalized Anxiety Disorder [GAD] – 7 Scale). Participants rated the items “feeling nervous, anxious or on edge” and “not being able to stop or control worrying” on the same scale as the PHQ-9. A sum score of 3 and more (range 0–6) indicated generalized anxiety with good sensitivity (86%) and specificity (83%)^26^.

The brief PHQ panic module was used to screen panic disorder. If at least two of the first four panic questions were answered with “yes”, caseness was defined^27^.

Social phobia was assessed with the German version of the Mini-Social Phobia Inventory (Mini-Spin)^28^ (e.g. “I avoid activities which put me in the center of attention”), rated from “not at all” (=0) to “extremely” (=3). Based on the cut-off score of 6 (range 0–12), the Mini-Spin has a good sensitivity (89%) and specificity (90%) to detect individuals with social anxiety disorder^28^.

Type D Personality was assessed with the German version of the DS-14^29^ with two 7-item subscales, negative affectivity (NA) and social inhibition (SI). It is answered on a 5-point- Likert scale from 0 (false) to 4 (true). The internal consistency of the DS-14 subscales is high (NA Cronbach’s α = 0.87, SI Cronbach’s α = 0.86). Type D personality is defined as a pattern of significant negative affectivity (NA ≥ 10) in conjunction with significant social inhibition (SI ≥ 10).

Socioeconomic status (SES) was defined according to Lampert *et al*.^30^ from 3 (lowest) to 21 (highest socioeconomic status) based on education, profession and income. Income was additionally calculated according to OECD.

Loneliness was assessed with the single item “I am frequently alone /have few contacts”. The item rated from 0 = “no, does not apply”, 1 = “yes it applies, but I do not suffer from it”, 2 = “yes, it applies, and I suffer slightly”, 3 = “yes, it applies, and I suffer moderately”, to 4 = “yes, it applies, and I suffer strongly”. Loneliness was defined combining 0 and 1 = no loneliness or distress; 2 = slight, 3 = moderate, and 4 = severe loneliness^31^.

Subjective health status was assessed by the question: “How would you describe your current health status?” (0 = worst; 100 = best health status).

Alcohol consumption and abuse was measured in gram per day; alcohol abuse was defined as daily consumption ≥40 mg for women and ≥60 mg for men.

The Short Questionnaire to Assess Health-Enhancing Physical Activity (SQUASH) evaluated physical activity^32^. It captures household, commuting, leisure time, work and school activities. Standing, lying, sleeping and sitting were classified as inactivity. Active sports were rated as the highest quartile of physical activity.

The presence of cardiovascular disease (CVD) was defined as history of self-reported myocardial infarction (MI), heart failure (HF), stroke, deep vein thrombosis (DVT), pulmonary embolism (PE), and peripheral arterial disease (PAD). Coronary heart disease was assessed by the question: “Were you diagnosed with a stenosis of your coronary vessels?” Cancer, COPD and asthma were assessed, correspondingly. Further, participants were asked, whether they had ever received the definite diagnosis of depressive disorder or any anxiety disorder by a physician.

Smoking was dichotomized into non-smoker and smoker. Non-smokers were defined as never and ex-smokers. Smokers were defined as occasional, i.e. <1 cigarette/day and regular smokers, i.e. >1 cigarette/day. Weight and height were measured according to a standardized, written manual. Obesity was defined as a body mass index (BMI) ≥ 30 kg/m². BMI was calculated (weight in kg divided by the square of height in meters)^33^. Diabetes was based on a blood glucose level of ≥126 mg/dl in the baseline examination after an overnight fast of at least 8 h, respectively a blood glucose level of ≥200 mg/dl after a fasting period <8 h or the diagnosis of diabetes by a physician. Dyslipidemia was defined as a low-density lipoprotein/high-density lipoprotein ratio of >3.5 or a diagnosis by a physician. Hypertension was diagnosed if the mean systolic blood pressure was ≥140 mm Hg (diastolic ≥90 mm Hg) in the 2nd and 3rd standardized measurement after 8 and 11 min of rest or if antihypertensive drugs were taken. FEV1 and FCV were assessed by spirometry^34^.

The New York Heart Association (NYHA) Functional Classification measures how much participants are limited during physical activity. NYHA 1 was defined as no symptoms and no limitation in ordinary physical activity, e.g. no shortness of breath when walking, climbing stairs, NYHA 2 was defined as mild symptoms (mild shortness of breath and/or angina) and slight limitation during ordinary activity.

Medications were registered at the study center by scanning the bar codes from the original drug packages of the participants. Three classes of antidepressants were entered as dichotomous variables (yes/no) as described by the ATC code: selective serotonin reuptake inhibitors (ATC N06AB), non-selective monoamine reuptake inhibitors (ATC N06AA) and other antidepressants (ATC N06AX).

### Statistical analysis

Descriptive analysis were performed as absolute and relative proportions for categorical data, means and standard deviations for continuous variables and median with interquartile range if not fulfilling normal distribution. Inference tests between depression groups (no/yes) were calculated with t-tests or Chi2 tests.

To analyze the relationship between depression and COPD, we used logistic regression analysis on the presence of depression (PHQ-9 > or <10).

To determine relations between depression, COPD and subjective health we used stepwise linear regression models. We also used stepwise linear regression models for men and women separately In the first model we included all sociodemographic, behavioral and psychological variables. In the second model we additionally included major somatic disorders (diabetes, CVD, COPD, cancer) in order to determine their additional contribution to somatic symptoms when the sociodemographic, behavioral and psychological variables are also in the equation.

All p-values should be regarded as a continuous parameter that reflect the level of statistical evidence and are therefore reported exactly. In addition, p-values are not adjusted for multiple testing. Hence all of the analyses are exploratory. Statistical analysis was carried out using R version 3.3.1.

## Results

### Sample characteristics

A total of 4.9% of the participants suffered from COPD or Asthma. Table 1 (baseline table) shows psychosocial and medical characteristics of participants with obstructive pulmonary disease compared to those without obstructive pulmonary disease.

**Table 1:** Demographical and clinical characteristics of participants with and without medicated COPD/asthma.

	All (*N* = 14294)	No COPD/asthma (*N* = 13591)	COPD/asthma (*N* = 703)	p
Age [y]	54.8 ± 11.1	54.6 ± 11.1	57.2 ± 10.9	**<0.0001**
Sex (% women)	49.6 (7086)	49.2 (6690)	56.3 (396)	**0.00027**
BMI [kg/m²]	26.6 (23.9/30.0)	26.5 (23.9/29.9)	27.8 (24.9/31.5)	**<0.0001**
**Spirometry**				
FEV1 (per SD)	297.3 ± 77.7	300.2 ± 76.5	241.8 ± 79.9	**<0.0001**
FVC	380 ± 95	383 ± 95	332 ± 94	**<0.0001**
COPD/Asthma (%)	4.9 (703)	0 (0)	100.0 (703)	**<0.0001**
NYHA ≥ 1 (%)	11.9 (1694)	10.1 (1378)	45.1 (316)	**<0.0001**
NYHA ≥ 2 (%)	2.5 (362)	2.0 (269)	13.3 (93)	**<0.0001**
**CVRFs, comorbid diseases**				
Diabetes (%)	8.9 (1275)	8.7 (1183)	13.2 (92)	**0.00013**
Obesity (%)	25.1 (3581)	24.6 (3339)	34.4 (242)	**<0.0001**
Smoking (%)	19.4 (2768)	19.2 (2609)	22.8 (159)	**0.024**
Hypertension (%)	49.3 (7050)	49.0 (6650)	56.9 (400)	**<0.0001**
Dyslipidemia (%)	43.9 (6268)	43.5 (5900)	52.6 (368)	**<0.0001**
FH of MI/Stroke (%)	22.1 (3160)	21.6 (2941)	31.2 (219)	**<0.0001**
CVD (%)	11.3 (1616)	10.8 (1473)	20.3 (143)	**<0.0001**
MI (%)	2.8 (399)	2.7 (363)	5.1 (36)	**0.00056**
Stroke (%)	1.7 (246)	1.6 (221)	3.6 (25)	**0.00053**
Cancer (%)	9.0 (1281)	8.7 (1184)	13.8 (97)	**<0.0001**
**Social and behavioral**				
SES	13.00 ± 4.43	13.06 ± 4.43	11.95 ± 4.35	**<0.0001**
Employment (%)	61.6 (8788)	62.3 (8447)	49.1 (341)	**<0.0001**
Income [€]	1625.00 (875.00/2625.00)	1875.00 (1125.00/2625.00)	1375.00 (875.00/2125.00)	**<0.0001**
Partnership (%)	81.3 (11618)	81.4 (11061)	79.2 (557)	0.15
Social support	21.00 (18.00/24.00)	21.00 (18.00/24.00)	21.00 (18.00/23.08)	**0.0067**
Loneliness (%)	10.5 (1493)	10.3 (1393)	14.3 (100)	**0.0012**
Alcohol (gram/day)	5.03 (0/17.60)	5.03 (0/17.60)	2.10 (0/14.07)	**0.0088**
Active sports (%)	49.0 (7008)	49.3 (6696)	44.4 (312)	**0.012**
Subjective health	67.11 ± 19.85	67.73 ± 19.48	55.19 ± 22.83	**<0.0001**
Ex-smoker (%)	34.9 (4983)	35.0 (4749)	33.5 (234)	0.46
Passive smoker (%)	3.7 (528)	3.7 (508)	2.8 (20)	0.26
Pack years	0.09 (0/3.21)	0.09 (0/3.14)	0.14 (0/5.66)	**0.012**
**Mental**				
PHQ9 ≥ 10 (%)	8.0 (1139)	7.5 (1025)	16.2 (114)	**<0.0001**
Suicidal thoughts (PHQ9) (%)	7.6 (1084)	7.5 (1006)	11.2 (78)	**0.00058**
History of depression (%)	12.0 (1707)	11.4 (1551)	22.2 (156)	**<0.0001**
Panic (%)	4.8 (662)	4.5 (595)	9.8 (67)	**<0.0001**
History of anxiety disorder (%)	7.2 (1031)	6.9 (942)	12.7 (89)	**<0.0001**
Social Phobia (Mini-Spin ≥6) (%)	7.3 (1044)	7.3 (982)	8.9 (62)	0.12
GAD2 ≥ 3 (%)	6.6 (936)	6.3 (846)	12.9 (90)	**<0.0001**
Type D (%)	23.7 (3377)	23.5 (3181)	28.0 (196)	**0.0071**
**PHQ9 classes**				
None (%)	64.7 (9183)	65.3 (8823)	51.6 (360)	n.a.
Minimal (%)	27.4 (3891)	27.2 (3667)	32.1 (224)	n.a.
Mild (%)	6.0 (856)	5.7 (769)	12.5 (87)	n.a.
Moderately (%)	1.5 (210)	1.4 (187)	3.3 (23)	n.a.
Severe (%)	0.5 (64)	0.4 (60)	0.6 (4)	n.a.
**Medication**				
Antidepressant (%)	5.6 (790)	5.3 (712)	11.1 (78)	**<0.0001**
SSRI (%)	1.9 (272)	1.8 (244)	4.0 (28)	**0.00031**
Anxiolytic (%)	0.9 (125)	0.8 (110)	2.1 (15)	**0.0013**
Benzodiazepine (%)	0.9 (121)	0.8 (107)	2.0 (14)	**0.0026**

Presented are means and standard deviations, rep. percentages; 1) median; Interquartile range; CVD = cardiovascular disease; MI = myocardial infarction; FH = family history; SES = socioeconomic status; PHQ9 = Patient Health Questionnaire Depression Score; GAD2 = Generalized Anxiety Score; SSRI = Selective Serotonin Reuptake Inhibitor.

Participants suffering from obstructive pulmonary disease were older and had a lower SES. They had clearly impaired lung function (FEV1, FVC), and almost half of the sample suffered from at least mild dypnea. There was an increased rate of cardiovascular risk factors, including obesity, smoking, a lack of active sports, hypertension, dyslipidemia and an increase of cardiovascular disease, diabetes, cancer and family history of MI, resp. stroke. While the great majority lived in a partnership, social support was reduced, and they reported more loneliness and type D behavior. There was no difference regarding social phobia.

As Table 1 shows, participants with obstructive pulmonary disease were older, more often female and had a lower SES. Mean pulmonary function was reduced based on FEV1 and FVC, and almost half suffered at least from mild dyspnea (13% from moderate to severe dyspnea). Obstructive pulmonary disease was associated with obesity, diabetes, slightly more current smoking (22.8% vs. 19.4%), less active sports and alcohol consumption, and more hypertension and dyslipidemia. There was also a high comorbidity with CVD including myocardial infarction (MI), stroke and cancer and a family history of MI, resp. stroke.

As psychosocial vulnerability factors, participants with obstructive pulmonary disease had lower SES, income and employment, and suffered from more loneliness and lower subjective health. As a psychobiological vulnerability factor, they had an increased rate of type D personality. They were more likely to have somatic comorbidities and more CVRFs compared to participants without COPD.

Table 2 shows multivariable logistic regression analyses with depression as dependent variable for all participants.

**Table 2:** Multivariable logistic regression model: predictors of depression (PHQ9 ≥ 10).

	(*N* = 13875, *N* = 1078 events)		
	OR	95% CI (L,U)	P
Age [5 y]	0.96	0.92, 1.01	0.17
Sex (% women)	1.72	1.41, 2.11	**<0.0001**
**Spirometry**			
FEV1 (per SD)	1.17	1.05, 1.32	**0.0055**
COPD/asthma (%)	1.71	1.30, 2.23	**0.00010**
NAHA ≥ 1 (%)	2.19	1.81, 2.64	**<0.0001**
**CVRFs, comorbid diseases**			
Diabetes (%)	1.14	0.89, 1.45	0.29
Obesity (%)	1.26	1.06, 1.49	**0.0063**
Smoking (%)	1.25	1.05, 1.48	**0.0087**
Hypertension (%)	0.99	0.84, 1.17	0.97
Dyslipidemia (%)	1.34	1.15, 1.57	**0.00016**
CVD (%)	1.30	1.03, 1.62	**0.020**
Cancer (%)	0.95	0.73, 1.22	0.70
**Social and behavioral**			
SES	0.98	0.96, 0.99	**0.034**
Partnership (%)	1.04	0.87, 1.24	0.63
Social support	0.90	0.88, 0.91	**<0.0001**
Loneliness (%)	2.83	2.38, 3.35	**<0.0001**
Alcohol abuse	0.75	0.46, 1.17	0.24
Active sports (%)	0.79	0.68, 0.91	**0.0019**
**Mental**			
Social Phobia (Mini-Spin ≥6) (%)	4.12	3.45, 4.92	**<0.0001**
Type D (%)	2.50	2.15, 2.91	**<0.0001**
Life events last 12 months (per 5 events)	1.74	1.53, 1.97	**<0.0001**

Note: 95% CI [L, U] = 95% confidence interval [lower value, upper value].

p: p-value. Depression: (PHQ-9 ≥ 10). Social Phobia: Mini-Spin ≥6. Life events last 12 months: per 5 events.

In multivariable logistic regression analyses, obstructive pulmonary disease was associated with increased depression (OR = 1.71; 95% CI = 1.30 to 2.24). Additional predictors were medical: FEV1 (1.18; 95% CI = 1.05 to 1.32) and dyspnea (NYHA > = 1) (OR = 2.19; CI = 1.82 to 2.64), demographic: sex (women) (OR = 1.73; 95% CI = 1.41 to 2.12), lower SES (OR = 0.98; 95%CI = 0.96 to 0.998). Cardiovascular risk factors such as active sports (OR = 0.79; 95% CI = 0.68 to 0.92), obesity (OR = 1.27; 95% CI = 1.07 to 1.49), smoking (OR = 1.26; 95% CI = 1.06 to 1.49) and dyslipidemia (OR = 1.35; 95% CI = 1.15 to 1.57) also predicted depression. Additional psychological risks were social phobia (OR = 4.13; 95% CI = 3.45 to 4.92), type D (OR = 2.51; 95% CI = 2.16 to 2.91), loneliness (OR = 2.83; 95% CI = 2.39 to 3.36) and life events last 12 months (per 5 events) (OR = 1.74; 95% CI = 1.53 to 1.98). CVD was a predictor; social support was protective (OR = 0.90; 95% CI = 0.89 to 0.92). Partnership, alcohol abuse, hypertension, cancer, diabetes were not associated with depression.

Table 3 presents the multiple linear regression on subjective health.

**Table 3:** Linear regression on subjective health (overall) (*N* = 12267).

	Estimate	95% CI [L, U]	P
Age [5 y]	−0.40	−0.57, −0.23	**<0.0001**
Sex (% women)	−0.12	−0.77, 0.51	0.70
**Spirometry**			
COPD/asthma (%)	−4.74	−6.30, −3.17	**<0.0001**
NYHA ≥ 1 (%)	−9.07	−10.1, −8.04	**<0.0001**
**CVRFs, comorbid diseases**			
Diabetes (%)	−3.80	−4.94, −2.67	**<0.0001**
Obesity (%)	−4.37	−5.12, −3.61	**<0.0001**
Smoking (%)	−2.68	−3.49, −1.88	**<0.0001**
Hypertension (%)	−3.36	−4.05, −2.68	**<0.0001**
Dyslipidemia (%)	−2.44	−3.10, −1.77	**<0.0001**
CVD (%)	−7.06	−8.08, −6.03	**<0.0001**
Cancer (%)	−4.52	−5.58, −3.45	**<0.0001**
**Social and behavioral**			
SES	0.29	0.21, 0.36	**<0.0001**
Partnership (%)	−1.04	−1.88, −0.20	**0.015**
Social support	0.47	0.37, 0.56	**<0.0001**
Loneliness (%)	−3.97	−5.07, −2.86	**<0.0001**
Alcohol abuse	−2.03	−3.83, −0.23	**0.027**
Active sports (%)	2.13	1.51, 2.76	**<0.0001**
**Mental**			
Depression (%)	−13.3	−14.6, −12.0	**<0.0001**
COPD/Asthma & Depression (%)	−19.9	−23.4, −16.4	**<0.0001**
Social Phobia (%)	−0.94	−2.19, 0.30	0.14
Type D (%)	−3.93	−4.69, −3.17	**<0.0001**
Life events last 12 months	−1.23	−1.86, −0.59	**0.00014**

Note: 95% CI [L, U] = 95% confidence interval [lower value, upper value].

p: p-value. Depression: (PHQ-9 ≥ 10). Social Phobia: Mini-Spin ≥6. Life events last 12 months: per 5 events.

As Table 3 shows, obstructive pulmonary disease and depression independently contributed to reduced subjective health in addition to sedentary behavior, smoking and comorbid somatic and mental disorders.

Subjective health was reduced in participants with obstructive pulmonary disease. In multivariable linear regression, depression was an additional factor, along with dyspnea, higher age, lower SES, a lack of a partnership, alcohol abuse, lack of active sports, obesity, smoking, type D, loneliness, lack of social support, and the presence of life events.

Supplementary Table S1 shows predictors of depression (PHQ9 ≥ 10) for men and women depicted separately.

## Discussion

The study presents population-based results on the association of depression, obstructive pulmonary disease (COPD or asthma) and subjective health in adults aged 35 to 74 years. In a large cohort of 15,010 participants from the community, we report data of 703 participants (4.9%) with obstructive pulmonary disease. Multivariable logistic regression analysis were performed in order to identify associations with depression above the presence of obstructive pulmonary disease, pulmonary functional decline and dyspnea. We observed a strong association between obstructive pulmonary disease and depression. Participants with COPD or asthma are more likely to suffer from depression and reduced subjective health. Depression and obstructive pulmonary disease both independently contributed to reduced subjective health.

These findings confirm the association between obstructive diseases and depression. A meta-analytical review^35^ identified that the prevalence of depressive symptoms was two times higher in people with COPD in comparison to controls. Zhang *et al*.^36^ found significantly higher depressive symptoms in patients with COPD (24,6%) than in controls (11,7%). In this study, the severity of COPD was not associated with the prevalence of depression. Similarly, the meta-analysis of Atlantis *et al*.^13^ suggested that COPD elevated the risk of depression (relative risk 1.69). The outcome of COPD, e.g. COPD exacerbation, was consistently increased by depression (relative risk 1.43).

A longitudinal study by Schneider *et al*. (n = 35,000 patients with COPD) with a follow-up of 10 years found 16.2 cases per 1000 person-years in the COPD group compared to 9.4 cases per 1000 person-years in the control group^37^. In this study, those with severe COPD were twice as likely to develop depression^13^ compared to patients with mild COPD.

The high comorbidity could be explained with symptoms present in COPD and depression. Both disorders are associated with withdrawal, anhedonia, fatigue, poor appetite, sleep disturbance, loss of concentrations and energy and reduced physical activity^38^.

However, in patients with COPD or asthma, the impact of social, biological, behavioral and psychological factors has not been thoroughly elucidated to date. It is important to clarify the impact of these factors on the risk of developing depression and reduced subjective health.

Smoking has long been known as an important cause of COPD in developed countries^6^. A positive association between smoking and depression has been reported^19,39–41^. Melvyn *et al*.^35^ reported that smoking status was not a significant moderator in predicting prevalence of depressive symptoms. This is not in line with the findings of this study. We found an association between smoking and prevalence of depression in participants with COPD or asthma. However, when analyzed separately for women and men we only found a significant association in women, but not in men (Supplementary Table S1).

Mevelyn *et al*.^35^ found that sex was not a significant moderator. Hanian *et al*.^9^ reported a significantly higher prevalence of depression in women suffering from COPD. In multivariable logistic regression analysis, we found an association between sex and prevalence of depression in participants with COPD or asthma.

Ottenheim *et al*.^42^ found in the oldest subjects, that COPD independently contributed to an increased risk of developing depressive symptoms. On the other hand, there is evidence, that age is not associated with higher depression prevalence in patients with COPD^7^. In this study, we found that participants suffering from obstructive pulmonary disease were older. In multiple regression analysis, however, age was not associated with higher depression prevalence in patients with COPD.

Findings of this study resonate with previous studies showing that the relationship between COPD or asthma and depression is indeed complex. Especially the degree of subjective impairment (dyspnea) played a significant adverse role, compounded by reduced objective function, social disadvantage, unhealthy behavior (smoking, no active sports, obesity, dyslipidemia), comorbid CVD, mental comorbidity (type D, social phobia), a lack of social support and loneliness and additional adverse life events in the previous years.

As we had expected, subjective health was also reduced in participants with obstructive pulmonary disease. In multivariable linear regression, depression was an additional factor, along with dyspnea, higher age, lower SES, a lack of a partnership, alcohol abuse, lack of active sports, obesity, smoking, type D, loneliness, lack of social support, and the presence of life events.

The complex interplay of biological, psychosocial and behavioral risk factors should be taken into account in the clinical care and guidance of COPD and asthma patients. In addition to the well-known risk profile of comorbid medical conditions and distress, a pattern of social disadvantage (lower SES) and behavioral risks needs to be attended in medical care. Promoting health behavior such as smoking cessation, adequate exercising and reducing weight may not only improve the somatic course of the disease but also reduce detrimental mental health consequences. Additionally, promotion of social support and integration may provide additional avenues of intervention.

### Strengths and limitations

Strengths refer to the large representative community sample and the comprehensive assessment of multiple sources integrating demographic, psychosocial, and behavioral determinants with mental and somatic comorbidities. The analyses covered participants who reported they were diagnosed and treated with COPD or asthma by a physician (and not limited to those who seek health care as in most previous studies). Yet, we were able to include a comprehensive range of biological variables including pulmonary function and symptoms (dyspnea) and comorbid disorders, social and psychological variables.

Limitations pertain to the fact that the medical diagnosis was self-reported, however, the functional measures show that there was significant objective and subjective impairment. Future analyses will test these findings in participants who are unaware of their obstructive pulmonary disease.

There is still a considerable need for prospective studies to continue investigation into the causality and temporal relationship between obstructive pulmonary disease and depressive symptoms. Participants with less severe complaints are more likely to take part in a community study. This might have led to selection bias towards oversampling those with the less severe depressive, respectively pulmonary symptoms. Also, we relied on data of validated questionnaires. We could not use expert clinical ratings of depression. Young (<35 years) and older participants (>75 years) did not take part in the examination. Prospective work should also target effective interventions (e.g. lifestyle, psychotherapy) to prevent and reduce depression in patients with COPD or asthma.

## Conclusion

Physical and mental health are strongly interrelated in medicated chronic obstructive pulmonary disease. There has been a strong increase of depression (16.2% vs. 7.5%) compared to participants without obstructive pulmonary disease. Complex underlying factors include sociodemographic (female sex, low SES), medical (FEV1, dyspnea), cardiovascular risk factors (smoking, obesity, dyslipidemia), and psychosocial factors (social phobia, type D, low social support, loneliness and stressful life events). The presence of obstructive lung disease and depression independently contributed to reduced subjective health. Promoting health behavior and social integration may not only improve the somatic course of the disease, but also mental health^43^.

## Supplementary information

Supplementary information

## Data Availability

For approved reasons, some access restrictions apply to the data underlying these findings. Data sets contain identifying participant information, which is not suitable for public deposition. Access to the local database is available upon request to the corresponding author.
