# Supplementary material for: Association between medicated obstructive pulmonary disease, depression and subjective health: results from the population-based Gutenberg Health Study
Source: Sci Rep. 2019 Dec 27;9:20252. doi: 10.1038/s41598-019-56440-9 (PMC6934802; doi:10.1038/s41598-019-56440-9)
Supplement: Supplementary file 1 — Supplementary information [file 41598_2019_56440_MOESM1_ESM.pdf]

Association between medicated obstructive pulmonary disease, depression and subjective health: results from the population-based Gutenberg Health Study

Jasmin Ghaemi Kerahrodi , MD,[1*] Elmar Brähler, PhD,[1] Jörg Wiltink, MD,[1] Matthias Michal, MD, [1] Andreas Schulz, PhD,[3] Phillip Wild, MD,[3,4,5] Thomas Münzel, MD,[2,5] Gerrit Toenges, PhD, [7] Karl Lackner, MD,[6,5] Norbert Pfeiffer, MD, [8] Manfred E. Beutel, MD [1]

[1] Department of Psychosomatic Medicine and Psychotherapy, University Medical Center of the Johannes Gutenberg University Mainz

[2] Center for Cardiology, Cardiology I, University Medical Center of the Johannes Gutenberg University, Mainz, Germany

[3] Preventive Cardiology and Preventive Medicine, Center for Cardiology, University Medical Center of the Johannes Gutenberg-University Mainz, Mainz, Germany

[4] Center for Thrombosis and Hemostasis, University Medical Center of the Johannes Gutenberg-University Mainz, Mainz, Germany

[5] DZHK (German Center for Cardiovascular Research), partner site RhineMain, Mainz

[6] Institute for Clinical Chemistry and Laboratory Medicine, University Medical Center of the Johannes Gutenberg University, Mainz, Germany

[7] Institute for Medical Biostatistics, Epidemiology and Informatics, University Medical Center of the Johannes Gutenberg-University Mainz, Germany

[8] Department of Ophthalmology, University Medical Center of the Johannes Gutenberg-University Mainz, Germany

* Corresponding author

Address correspondence to: Jasmin Ghaemi Kerahrodi, Department of Psychosomatic Medicine and Psychotherapy, University Medical Center, Johannes Gutenberg-University, Untere Zahlbacher Straße 8, 55131 Mainz, Germany, Email: jasmin.ghaemi@unimedizin-mainz.de, Telephone: +49 6131 17-2912 / Fax: +49 6131 17-6688

Supplementary table S1: Multiple logistic regression model: predictors of depression (PHQ9≥10) for men and for women

	Men (*N* = 7032, *N* = 429 events)			Women (*N* = 6825, *N* = 649 events)		
	OR	95% CI (L,U)	P	OR	95% CI (L,U)	P
Age [5y]	0.963	0.895, 1.036	.31	0.969	0.909, 1.033	0.33
**Spirometry**						
FEV1 (per SD)	1.151	0.985, 1.345	.077	1.273	1.061, 1.529	**0.0094**
COPD/Asthma (%)	1.935	1.232, 2.970	**.0032**	1.604	1.129, 2.250	**0.0072**
NYHA≥1 (%)	2.316	1.677, 3.180	**<.0001**	2.213	1.751, 2.790	**< 0.0001**
**CVRFs, comorbid diseases**						
Diabetes (%)	1.139	0.799, 1.602	.46	1.105	0.778, 1.550	0.57
Obesity (%)	1.300	1.006, 1.676	**.044**	1.240	0.991, 1.545	0.058
Smoking (%)	1.273	0.981, 1.645	.067	1.277	1.015, 1.601	**0.036**
Hypertension (%)	1.016	0.795, 1.298	.90	1.006	0.808, 1.250	0.96
Dyslipidemia (%)	1.492	1.175, 1.901	**.0011**	1.261	1.022, 1.553	**0.030**
CVD (%)	1.238	0.895, 1.697	.19	1.307	0.946, 1.784	0.098
Cancer (%)	0.953	0.602, 1.459	.83	0.960	0.699, 1.302	0.80
**Social and behavioral**						
SES	0.986	0.960, 1.013	.30	0.975	0.950, 1.000	0.050
Partnership (%)	1.880	1.383, 2.581	**<.0001**	0.784	0.636, 0.969	**0.023**
Social support	0.894	0.869, 0.921	**<.0001**	0.904	0.883, 0.924	**< 0.0001**
Loneliness (%)	3.386	2.551, 4.483	**<.0001**	2.636	2.124, 3.266	**< 0.0001**
Alcohol abuse	0.879	0.485, 1.501	.65	0.584	0.243, 1.227	0.19
Active sports (%)	0.615	0.482, 0.783	**<.0001**	0.915	0.757, 1.107	0.36
**Mental**						
Social Phobia (Mini-Spin≥6) (%)	4.452	3.343, 5.910	**<.0001**	4.065	3.237, 5.099	**< 0.0001**
Type D (%)	3.256	2.575, 4.120	**<.0001**	2.159	1.773, 2.627	**< 0.0001**
Life events last 12 months (per 5 events)	1.577	1.293, 1.920	**<.0001**	1.857	1.5662.200	**< 0.0001**

Note: 95% CI [L, U] = 95% confidence interval [lower value, upper value].

p: p-value. Depression: (PHQ-9≥10). Social Phobia: Mini-Spin≥6. Life events last 12 months: per 5 events.
